# Dual regulation of anthocyanins and proanthocyanidins by *PpBL* and its linkage to acidity in peach

**DOI:** 10.1186/s43897-026-00255-6

**Published:** 2026-09-01

**Authors:** Hongyang Xing, Jiaqi Fan, Guizhi Li, Ke Cao, Yong Li, Gengrui Zhu, Weichao Fang, Changwen Chen, Xinwei Wang, Jinlong Wu, Lirong Wang

**Affiliations:** 1 National Key Laboratory for Germplasm Innovation & Utilization of Horticultural Crop, The Key Laboratory of Biology and Genetic Improvement of Horticultural Crops (Fruit Tree Breeding Technology), Zhengzhou Fruit Research Institute, Chinese Academy of Agricultural Sciences, Ministry of Agriculture and Rural Affairs, 500 meters south of the intersection of Hanghai Road and Weilai Road, Zhengzhou, Guancheng Hui District 450009 China; 2 Zhongyuan Research Center, Chinese Academy of Agricultural Sciences, No. 28 Hongqiqu Road, East Hall, Building 2, Chuangzhi Gongyuan, Henan Testing and Inspection Industry Park, Pingyuan Demonstration Zone, Xinxiang, 453599 China; 3 Western Research Institute, Chinese Academy of Agricultural Sciences, No. 195 Ningbian East Road, Changji, Changji Hui Autonomous Prefecture, Changji, 831100 China

**Keywords:** Anthocyanin, Proanthocyanidin, Fruit acidity, QTL mapping, *PpBL*

## Abstract

Red-fleshed peach exhibits attractive anthocyanin (AN) pigmentation but is often accompanied by undesirable proanthocyanidin (PA)-derived astringency and high fruit acidity (FA), limiting its commercial value. The genetic mechanisms underlying this co-occurrence remain unclear. Using an F_1_ population derived from ‘wen30’ and ‘wen48’, we identified co-localized QTLs for AN, PA, and FA within a 0.32–4.74 Mb region on chromosome 5. Integrating transcriptomics with Y1H, EMSA, and dual-luciferase assays, we demonstrated that *PpBL* functions as a key regulator of both AN and PA accumulation. *PpBL* directly activates *PpUGT73C3*, independently of *PpMYB10.1*, to promote AN biosynthesis, and enhances PA accumulation by directly upregulating *PpLAR* and *PpANR*; *PpUGT73C3* also contributes to PA accumulation via an unclear mechanism. In contrast, FA is not directly regulated by *PpBL* but is associated with a high-acidity *PpTST1* allele tightly linked to a blood-TE insertion in the *PpBL* promoter. The haplotype carrying both the insertion and *PpUGT73C3* coding variants confers elevated AN and PA but also increased FA due to linkage drag. These findings reveal pleiotropic control of color and astringency and a linkage-based association with acidity, providing insights for peach breeding.

**Supplementary Information:**

The online version contains supplementary material available at https://doi.org/10.1186/s43897-026-00255-6.

## Core

We identified a co-localized QTL on chromosome 5 that governs both anthocyanins and proanthocyanidins accumulation. We further demonstrated that *PpBL* acts as a master regulator of these two traits. Importantly, we provided a genetic explanation for the observed correlation between red flesh and high acidity: the *PpBL* locus is tightly linked to—but does not directly regulate—the acidity-determining gene *PpTST1*.

## Gene & accession numbers

The accession numbers are: PpBL (Prupe.5G006200), PpUGT73C3 (Prupe.5G022600), PpLAR (Prupe.1G305100), PpANR (Prupe.4G029700), PpMYB10.1 (Prupe.3G163100), PpTST1 (Prupe.5G006300), PpANS (Prupe.5G086700), PpNAD-MDH1 (Prupe.8G157300), PpALMT-9 (Prupe.1G308300).

## Introduction

Rising consumer demand for premium fruits with rich flavor and vibrant color highlights the importance of appearance in determining the market value of fruit crops. Consumers tend to favor red fruits for their visual appeal (Liu et al. 2017). Anthocyanin (AN), the key pigments responsible for red coloration in fruits, not only enhance aesthetic quality but also provide antioxidant benefits that are beneficial to human health (Wang and Mazza 2002; Xia et al. 2006; Martin et al. 2011; Asakura et al. 2024). In response to this market preference, breeders are increasingly focusing on developing new red-fleshed varieties.

The red coloration in peaches results from the accumulation of AN, which along with other polyphenols abundant in red-fleshed peaches confers strong antioxidant properties (Zhou 2014; Zhao et al. 2024). AN biosynthesis in peaches occurs through the phenylalanine and flavonoid pathways, a mechanism conserved across species such as apple (Xing et al. 2023), strawberry (Mao et al. 2022), and kiwifruit (Liu et al. 2022). Two major loci, dominant blood-flesh (*DBF*) and recessive blood-flesh (*bf*), have been identified as key genetic determinants of the red-flesh trait in peaches. The *DBF*-encoded gene, *PpBL*, located at the beginning of Chr5, interacts with *PpNAC1* to form a dimer that enhances the expression of P*pMYB10.1*, thereby promoting AN accumulation (Zhou et al. 2015). Conversely, the recessive *bf* locus, mapped to Chr4: 4.21–4.52 Mb, controls red-flesh formation in homozygous genotype (Shen 2013). Additionally, multiple structural genes contribute to AN biosynthesis and regulation, including *PpDFR* (Zhou et al. 2020), *PpGST1* (Lu et al. 2021; Zhao et al. 2020), *PpANS*, *PpUFGT* (Tsuda et al. 2004). Among them, UDP-glucose:flavonoid 3-O-glucotransferases (UFGTs) act as the final enzymatic step, stabilizing AN into pigmented form and thus serving as a key determinant of fruit color. In peach, *PpUGT78A1* and *PpUGT78A2* have been shown to mediate AN glycosylation (Cheng et al. 2014). Furthermore, several transcription factors, including *PpMYB10.1* (Tuan et al. 2015), *PpHYH* (Zhao et al. 2022), *PpMYB6*, *PpbHLH35* (Wang et al. 2022), and *PpSPL1* (Zhou et al. 2015), have been identified as important regulators of AN synthesis in peach fruits.

Color is not an isolated agronomic trait, as the genes governing color often exert pleiotropic effects on other characteristics. For instance, in pear, *PyBBX24* promotes AN biosynthesis while reducing bioactive gibberellic acid (GA) levels, leading to plant dwarfism (Yang et al. 2024). In *Brassica napus*, the dominant quantitative trait locus (QTL) *cqSC-A09*, which controls yellow seed coat formation, also affects seed oil and fiber content. The dominant allele increases oil content while reducing pigment and fiber content (Chao et al. 2022). Similarly, in red-fleshed peaches, high AN content is often accompanied by elevated levels of PA and FA, contributing to stronger astringency and sourness (Yan J et al. 2014; Xu et al. 2022). PA content is primarily influenced by structural genes such as *PpANR* and *PpLAR*, along with transcription factors including *PpMYBPA1*, *PpMYB7*, *PpMYB18*, and *PpbZIP5* (Ravaglia et al. 2013; Zhou et al. 2015; Yan J et al. 2018; Zhou et al. 2019). Since both AN and PA are derived from the flavonoid pathway, specific transcription factors can coordinately regulate their biosynthesis. For example, in grape, *VvMYB86* modulates the metabolic flux between the PA and AN branches by upregulating *VvLAR* to promote PA synthesis while downregulating *VvANS* and *VvUFGT* to suppress AN production (Cheng et al. 2020). In apple, *MdNAC52* indirectly regulates both AN and PA synthesis by binding to the promoters of *MdMYB9* and *MdMYB11*, and directly activates PA synthesis via the *MdLAR* and *MdANR* promoters (Mao et al. 2021). Although *PpMYB18* has been associated with AN and PA content in peach, no functional variants have been identified, suggesting it may not be the key regulator of AN and PA metabolism in red-fleshed peaches. Thus, the core regulators remain to be identified.

FA is another key factor influencing the flavor of red-fleshed peaches, primarily determined by the accumulation of various organic acids, such as malate, citrate, quinate, and tartrate, among which malate and citrate are the most abundant (Etienne et al. 2013). Previous studies have reported higher levels of malic acid and titratable acid in red-fleshed fruit (Cui 2014; Xu et al. 2022; Chen et al. 2025). The major gene controlling peach fruit acidity, designated as the D locus, is located at the proximal end of chromosome 5, and low acidity is the dominant trait (Cao et al. 2019). Through genome-wide association studies (GWAS) and comparative transcriptome analysis, *PpTST1*, encoding a tonoplast sugar transporter, has been identified as a strong candidate gene for organic acid accumulation (Wang et al. 2022). Notably, *PpBL*, the key gene controlling red-flesh coloration, has also been strongly associated with malate accumulation (Chen et al. 2025), suggesting a potential genetic link between coloration and fruit acidity. However, whether this link reflects direct regulation by *PpBL* or tight genetic linkage remains to be elucidated.

In summary, although red-fleshed peaches exhibit bright coloration and are rich in health-beneficial antioxidants, they often suffer from poor flavor. However, comprehensive studies integrating color, astringency, and acidity in peach populations are lacking. Consequently, the genetic basis underlying their co-segregation, specifically whether it arises from pleiotropy or genetic linkage, remains unclear. The core genes coordinately regulating color, astringency, and acidity have yet to be identified.

In this study, an F_1_ segregating population was developed by crossing two red-fleshed peach cultivars, ‘wen30’ and ‘wen48’. Phenotypic analysis revealed significant positive correlations among AN, PA, and FA levels. Using a high-density genetic map, we performed QTL mapping for the three traits and identified co-localized QTLs on Chr5. Integrated transcriptome analysis and gene functional annotation identified *PpBL* and *PpUGT73C3* as candidate genes. We further demonstrated that *PpBL* directly activates *PpUGT73C3* to drive AN biosynthesis and promotes PA synthesis via *PpLAR/PpANR*. Additionally, *PpUGT73C3* also promotes PA accumulation, though the mechanism is unclear. Notably, the long terminal repeat-retrotransposon (blood-TE) insertion in the promoter region of *PpBL* and the non-synonymous mutations in *PpUGT73C3* coding sequence (CDS) were closely associated with AN, PA, and FA contents. Although *PpBL* does not directly regulate FA, the blood-TE insertion is in genetic linkage with SNPs in *PpTST1*, a key gene controlling organic acid accumulation. In summary, this work identifies key genes coordinately regulating AN and PA levels in red-fleshed peach and clarifies that their co-segregation with FA results from genetic linkage with *PpTST1*. These findings provide valuable molecular tools for breeding red-fleshed peaches with enhanced color and balanced flavor.

## Results

### Phenotypic analysis

**Fig. 1:**
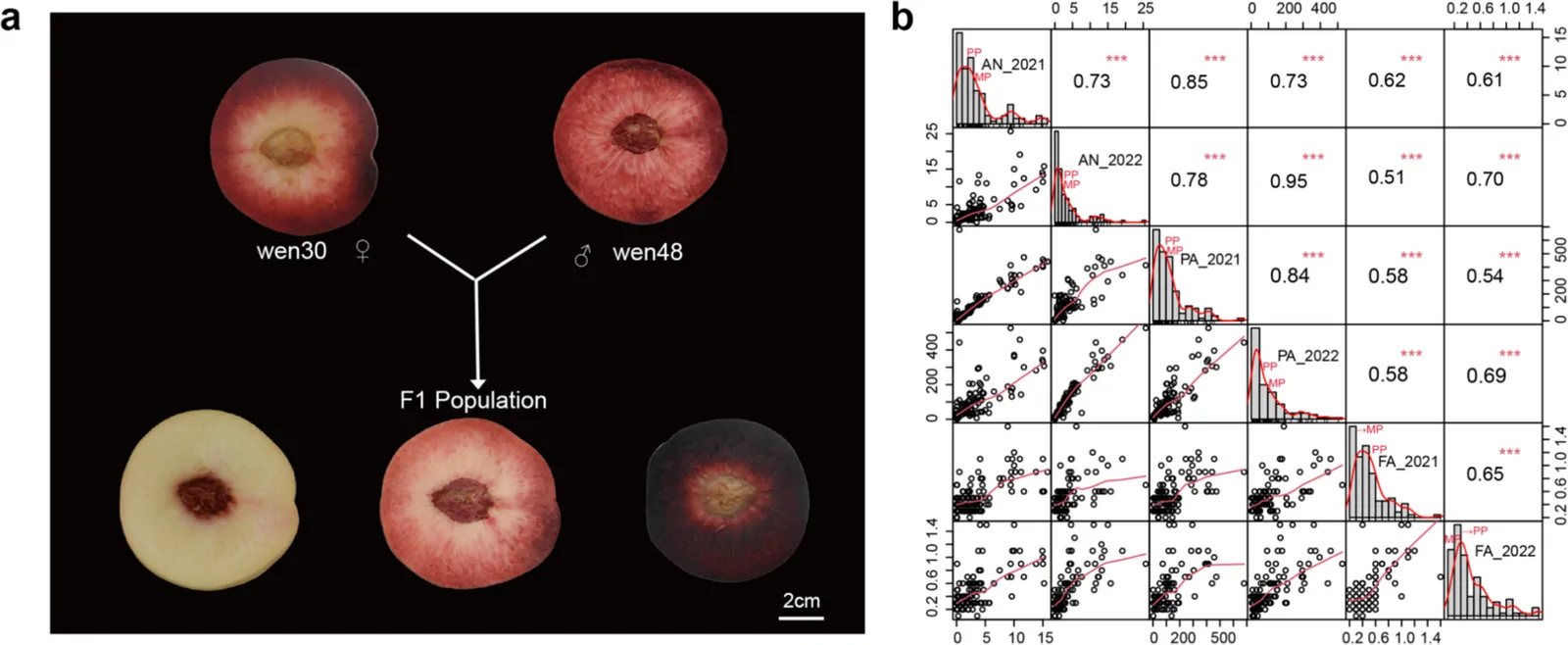
Fruit traits analysis of the F1 population. **a** The fruit color of ‘wen30’, ‘wen48’, and the F1 population. Scale bar: 2 cm. **b** Phenotypic data distribution and correlation coefficients among fruit traits in the F1 population. The diagonal panels show the distribution of AN_2021, AN_2022, PA_2021, PA_2022, FA_2021, FA_2022, where AN stands for anthocyanin, PA for proanthocyanidins, and FA for fruit acidity. PP indicates the paternal parent, and MP indicates the maternal parent. The upper right panels show the correlation coefficients and significance levels. The lower left panels show bivariate scatter plots with fitted lines. Statistical significance: ***P < 0.001

A total of 152 F_1_ individuals were categorized into red- and white-fleshed types, with an observed segregation ratio of 123 red to 29 white. This ratio conformed to the expected Mendelian 3:1 segregation (χ2 = 3.45), indicating that the red flesh trait is dominant and controlled by a single gene. The traits AN, PA, and FA exhibited transgressive inheritance in the F_1_ population, with consistently high distributions and variation rates across two consecutive years (Fig. 1a; Table 1). Specifically, in 2021, the AN content was 2.86 μmol·g^−1^ DW in the paternal parent and 3.29 μmol·g^−1^ DW in the maternal parent, while the F1 population exhibited a range of 0.01 to 15.81 μmol·g^−1^ DW. The PA content was 126.42 mg·g^−1^ DW in the paternal parent and 113.43 mg·g^−1^ DW in the maternal parent, whereas the offspring values ranged from 0.74 to 579.39 mg·g^−1^ DW. For FA, parental values were 0.51% and 0.27%, compared to an offspring range of 0.21% to 1.55%. Similar transgressive expression was observed in 2022.

**Table 1:** Summary statistics analysis of phenotypes for AN, PA and Acid in the F1 progeny and parents for two years

Trait	Year	Mean of paternal parent	Mean of maternal parent	F1				H^2^
				Range	Mean ± SD	COV(%)	Skew	
AN (μmol·g^-1 ^DW)	2021	2.86	3.09	0.01-15.81	3.72±3.94	105.91	1.47	-
	2022	2.40	2.45	0.01-15.79	3.31±3.97	119.93	1.65	82%
PA (mg·g^-1 ^DW)	2021	126.42	113.43	0.74-579.39	127.83±122.97	96.20	1.47	-
	2022	80.62	110.54	0.97-526.36	102.89±111.68	108.45	1.72	86%
FA	2021	0.51	0.27	0.21-1.55	0.53±0.25	47.17	1.24	-
(%)	2022	0.31	0.20	0.11-1.52	0.48±0.30	62.50	1.51	65%

Furthermore, the H^2^ of AN, PA, and FA were 82%, 86%, and 65%, respectively (Table 1), indicating a strong genetic basis for these traits. Phenotypic correlation analysis revealed positive associations among AN, PA, and FA. Pearson's correlation coefficients between AN and PA were 0.85 (*P* < 0.001) in 2021 and 0.95 (*P* < 0.001) in 2022, while those between AN and FA were 0.62 (*P* < 0.001) in 2021 and 0.70 (*P* < 0.001) in 2022 (Fig. 1b).

### High-density genetic map construction and QTL analysis

Sequencing was performed on two red-fleshed parents (‘wen30’ and ‘wen48’) and 152 hybrid progeny, yielding 27,583,960, 23,741,062, and 3,287,219,706 clean reads, respectively (Table S2). The mapping rates were 81.42% and 85.85% for the parental lines, and 84.76% for the progeny population. After filtering out the low quality and non-dimorphic SNP loci, a total of 394,679 high-quality SNPs were obtained. Using Lep-MAP3, we constructed a genetic map spanning 530.690 cM, which consisted of 1,339 bins distributed across 8 linkage groups, with an average inter-marker distance of 0.396 cM (Table 2; Fig. S1).

**Table 2:** Summary of the high-density genetic map of ‘wen30’ × ‘wen48’ F1 population

Chromosomes	Length (cM)	Number of bins	Average distance (cM)
Chr1	98.462	268	0.367
Chr2	63.285	163	0.388
Chr3	63.276	170	0.372
Chr4	61.730	171	0.361
Chr5	50.047	109	0.459
Chr6	65.180	123	0.530
Chr7	62.040	173	0.359
Chr8	66.670	162	0.412
Average	66.336	167.375	0.396
Total	530.690	1339	-

**Fig. 2:**
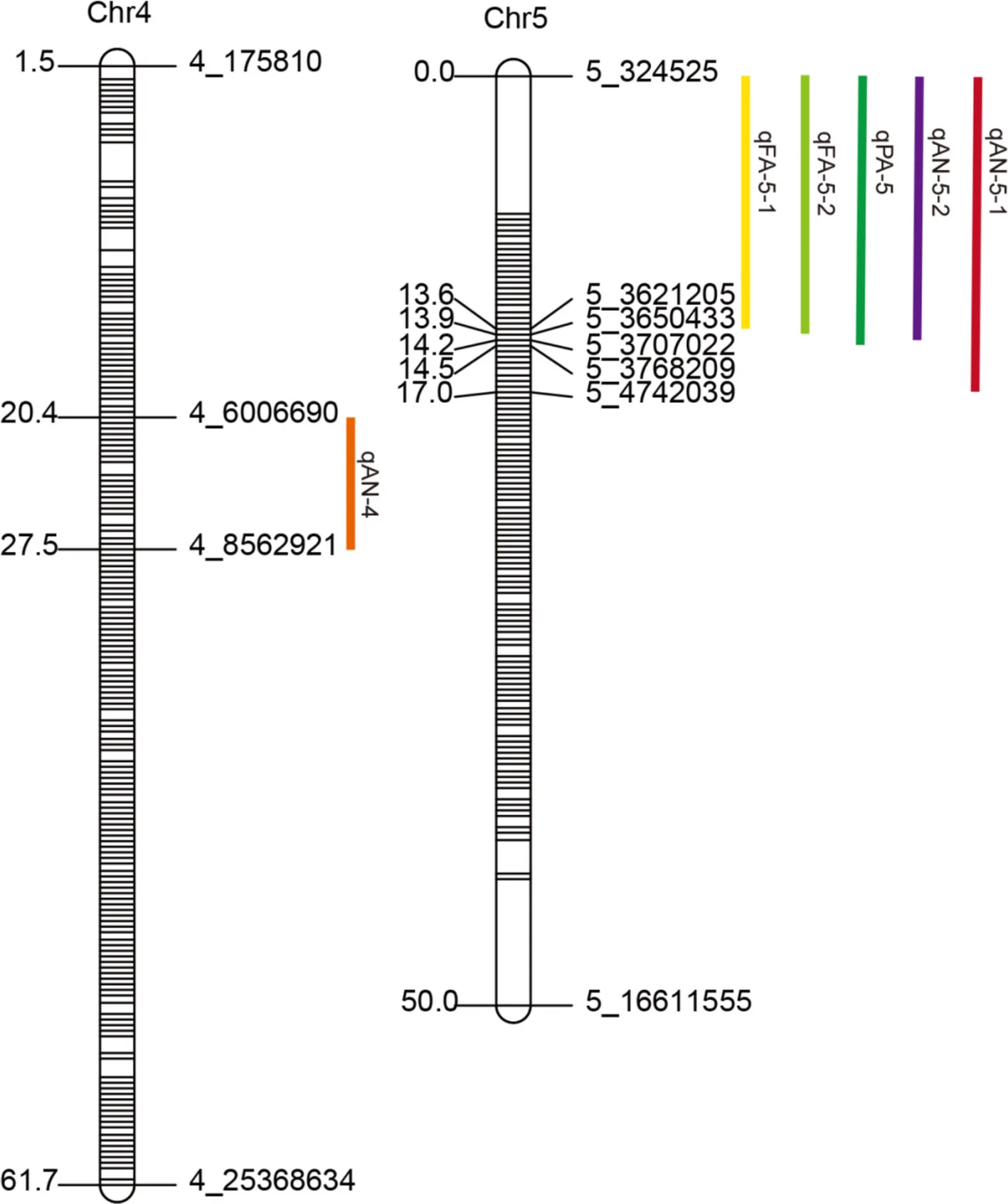
Genetic linkage map and QTL locations. The numbers on the left of the map represent the positions on the genetic map, while those on the right indicate the corresponding markers. QTLs are named starting with 'q', followed by the trait abbreviation (AN stands for anthocyanin, PA for proanthocyanidins, FA for fruit acidity), and then the chromosome number

Based on this genetic map, we performed QTL analyses for AN, PA, and FA (Fig. 2; Fig. S2; Table S3). Two QTLs associated with AN were detected on Chr4 and Chr5, explaining 15.88% and 57.77% of the phenotypic variance (PVE), respectively. Specifically, *qAN-4* was mapped to Chr4: 20.5–27.5 cM with a LOD of 9.37, while *qAN-5* was located at Chr5: 3.6–16.7 cM with LOD scores ranging from 25.23 to 26.28. A QTL for PA was identified on Chr5 between 0–14.4 cM, with LOD scores of 20.47–30.05 and PVE values of 58.46%–65.69%. Similarly, a QTL for FA was also found on Chr5 (0.6–13.9 cM), showing LOD scores of 23.01–31.60 and PVE value of 55.68%–67.68%.

Notably, the QTL intervals for AN, PA, and FA co-localized within the Chr5: 3.7–13.6 cM region. This overlapping segment was therefore considered a stable QTL interval and was found to encompass 260 genes.

### Identification of candidate genes *PpBL* and *PpUGT73C3*

**Fig. 3:**
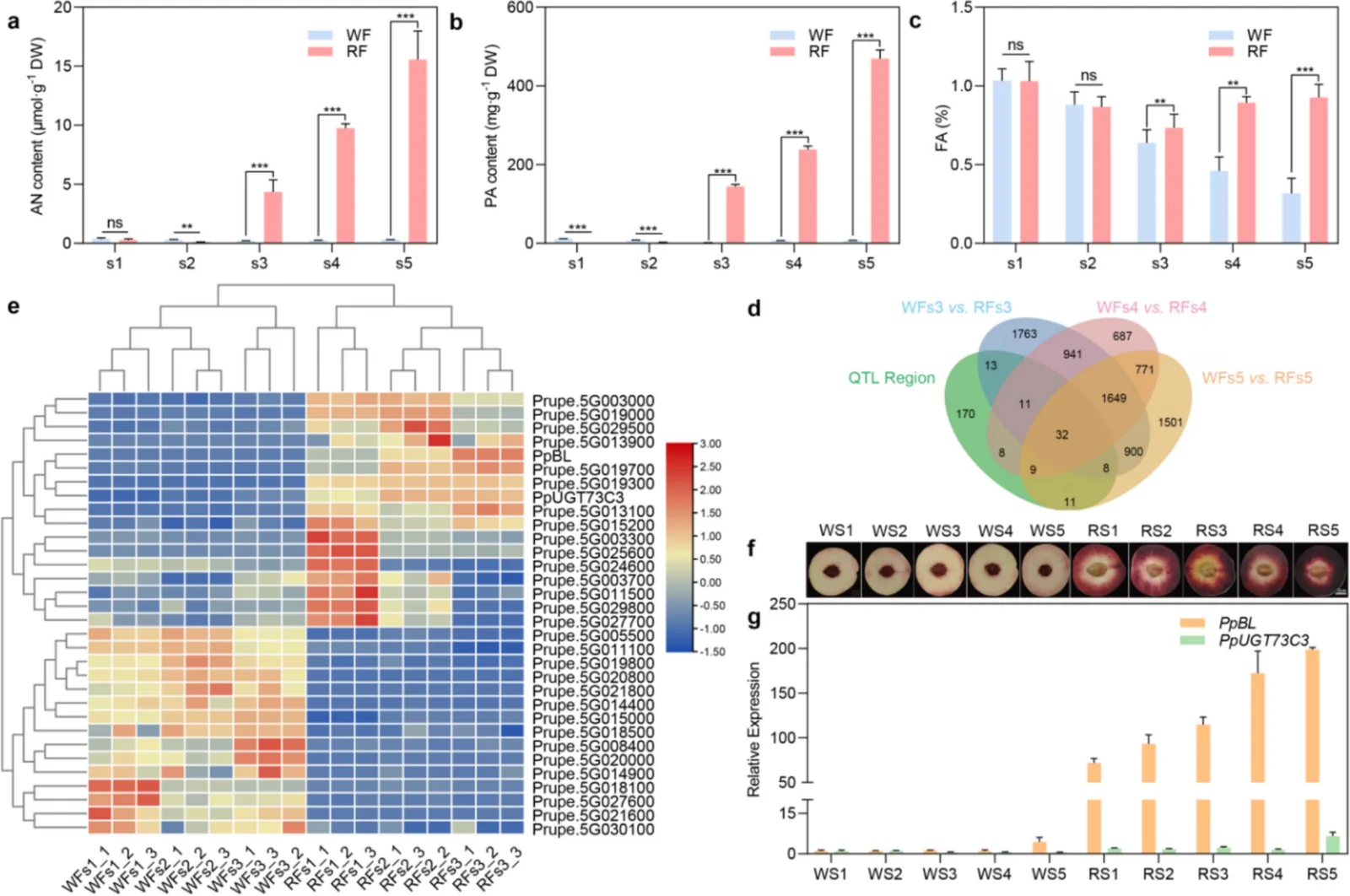
Screening of candidate genes based on QTL mapping and transcriptome analysis. **a**–**c** AN, PA, and FA concentrations at different fruit developmental stages. AN stands for anthocyanin, PA for proanthocyanidins, FA for fruit acidity, WF for white-fleshed peach, and RF for red-fleshed peach. Statistical significance: ***P < 0.001; **P < 0.01; ns, not significant. **d** Venn diagram of the QTL region and RNA-seq data. **e** Heatmap of differentially expressed genes (DEGs) between WF and RF within the candidate QTL region. **f** Phenotypes of five white-fleshed samples (WS) and five red-fleshed samples (RS). Scale bar: 2 cm. **g** Relative expression of *PpBL* and *PpUGT73C3* in five white-fleshed and five red-fleshed samples

To identify genes regulating the synthesis of AN, PA, and FA, white-fleshed (WF) and red-fleshed (RF) peaches were selected from the F1 progeny. The contents of AN, PA, and FA were measured at 40, 70, 80, 90, and 100 days after anthesis, corresponding to developmental stages s1 to s5. In WF fruits, AN and PA levels remained low throughout all developmental stages. In contrast, RF fruits exhibited a rapid increase after s2, with significantly higher concentrations than those in WF. Specifically, during s3 to s5, AN content in RF was 21**-**, 37**-**, and 52**-**fold higher than in WF, while PA content was 111**-**, 37**-**, and 72**-**fold higher, respectively (Fig. 3a–b; Table S4). FA levels in WF decreased from s1 to s5. In RF, FA levels gradually decreased from s1 to s3 but increased during later stages of fruit ripening (Fig. 3c). During s3–s5, FA content in RF was 1.18**-**, 1.93**-**, and 2.97**-**fold higher than in WF, respectively (Table S4).

Based on these observations, flesh samples from WF and RF at s3, s4, and s5 were selected for RNA sequencing (Table S5). Comparative transcriptome analysis between WF and RF at each stage (WFs3 *vs.* RFs3, WFs4 *vs.* RFs4 and WFs5 *vs.* RFs5) identified 806 up-regulated and 448 down-regulated differentially expressed genes (DEGs) (Fig. S3). Among these, 32 DEGs were located within the QTL interval (Fig. 3d–e; Table S6). Notably, this set included *PpBL* (*Prupe.5G006200*), a known regulator of anthocyanin biosynthesis in red-fleshed peach (Zhou et al. 2015), and *PpUGT73C3* (*Prupe.5G022600*), a glycosyltransferase involved in anthocyanin glycosylation. For qRT-PCR validation, five white-fleshed samples (WS) and five red-fleshed samples (RS) were randomly selected from the F_1_ progeny (Fig. 3f). Both *PpBL* and *PpUGT73C3* showed higher expression levels in RS compared to WS (Fig. 3g). It is also noteworthy that *PpTST1*, a gene reported to regulate organic acid accumulation in fruits, falls within our QTL interval, although it was not identified as a DEG.

### Genotype diversity analysis of candidate genes

**Fig. 4:**
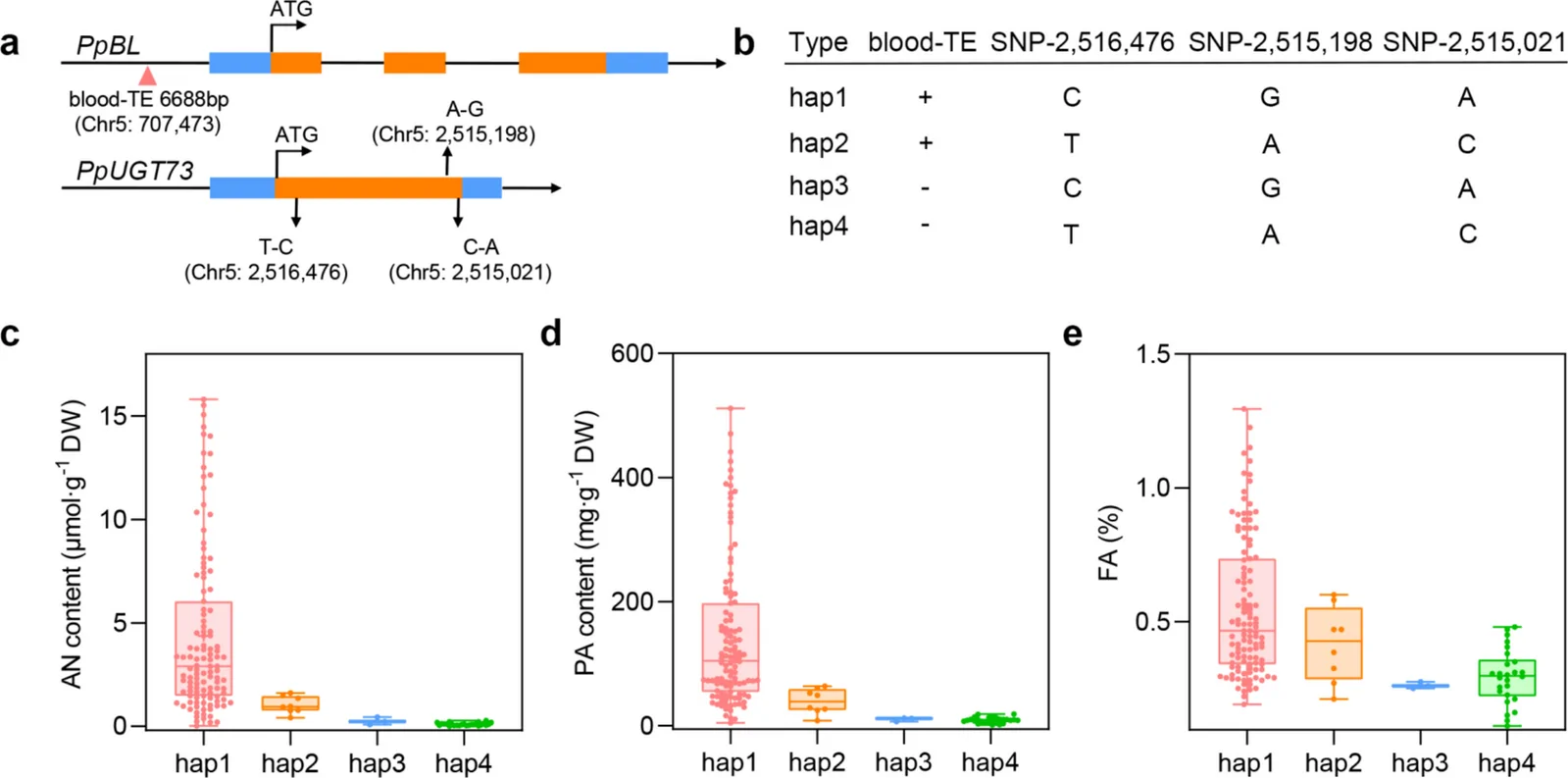
Genotypic diversity analysis of candidate genes. **a** Variants of *PpBL* and *PpUGT73C3*. **b** Four haplotypes containing blood-TE and SNP variations. **c** Genotypes of different haplotypes for anthocyanin (AN). **d** Genotypes of different haplotypes for proanthocyanidins (PA). **e** Genotypes of different haplotypes for fruit acidity (FA)

We then quantified the variants of *PpBL* and *PpUGT73C3* in both the parental and F_1_ progeny lines. The 6688-bp blood-TE in the promoter region of *PpBL* has previously been reported to co-segregate with red flesh coloration (Wang et al. 2024). Therefore, primers were designed to detect the distribution of blood-TE in the parents and the F1 progeny. The results revealed a heterozygous insertion of the transposon in the parental lines and a segregation ratio of 27:90:36 in the F1 progeny (Table S7). Furthermore, three non-synonymous mutations were identified in the CDS of *PpUGT73C3*, which showed strong linkage with the blood-TE in the *PpBL* promoter region (Fig. 4a). Based on these tightly linked variants, the F1 progeny were categorized into four haplotypes (Hap) (Fig. 4b). Hap1 and hap2 were the predominant haplotypes, represented by 115 and 24 individuals, respectively (Fig. 4c–e). The contents of AN, PA, and FA in hap1 were significantly higher than in the other haplotypes, indicating its potential value for breeding in red-fleshed peaches.

### Characterization of PpUGT73C3 enzyme activity

The allele of *PpUGT73C3* harboring three non-synonymous mutations was designed as *Ppugt73c3*. We then performed sequence alignment and phylogenetic analysis of *PpUGT73C3* and its allele *Ppugt73c3*, along with functionally characterized UGT homologs, including.

**Fig. 5:**
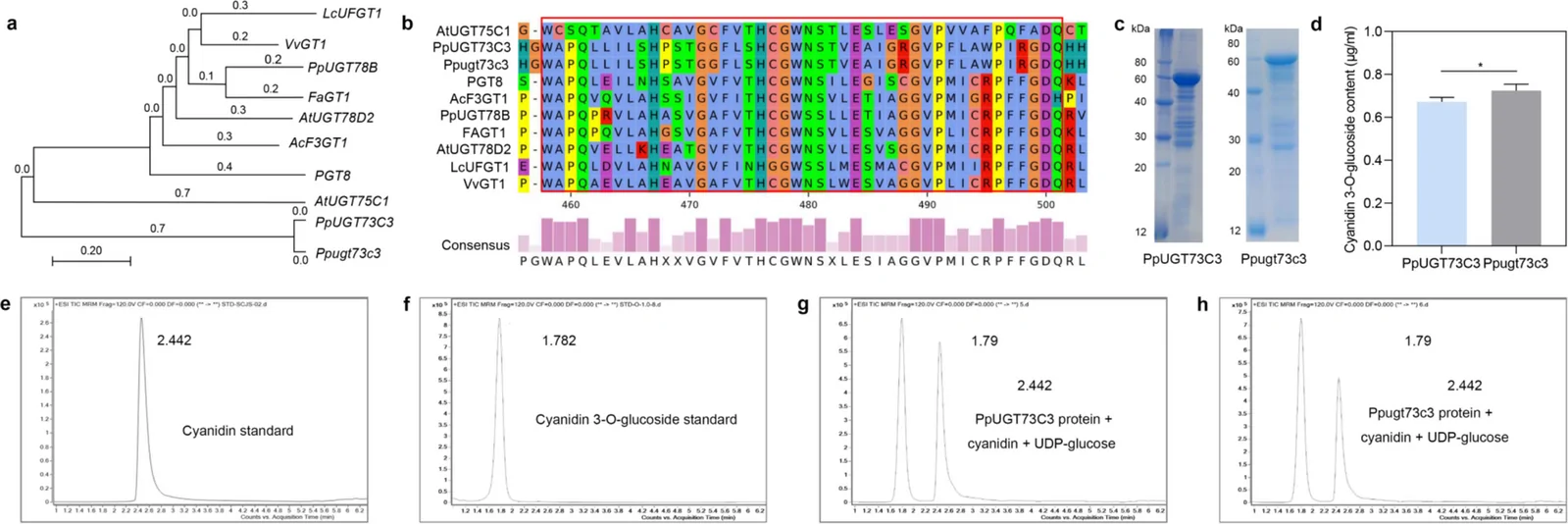
Sequence and enzymatic activity analysis of *PpUGT73C3* and its allele *Ppugt73c3*. **a** Phylogenetic tree analysis of *PpUGT73C3* sequences in peach and other species. **b** Alignment of PpUGT73C3 proteins in peach and other species. The red box indicates a conserved sequence consisting of 44 amino acids, namely the PSPG box. **c** Analysis of purified PpUGT73C3 and Ppugt73c3 recombinant proteins using 10% SDS-PAGE. **d** Comparison of enzyme activity in vitro*.* Values are means ± SD for three replicates. Statistical significance: *P < 0.05. **e** LC–MS analysis of cyanidin standard. **f** LC–MS analysis of cyanidin 3-O-glucoside standard. **g** LC–MS analysis of the reaction mixture containing PpUGT73C3, cyanidin, and UDP-glucose. **h** LC–MS analysis of the reaction mixture containing Ppugt73c3, cyanidin, and UDP-glucose

*LcUGFT1* from lychee (Li et al. 2016), *VvGT1* from grapevine (Offen et al. 2006), *PpUGT78B* from peach (Cheng et al. 2014), *FaGT1* from strawberry (Griesser et al. 2008), *AtUGT78D2* and *AtUGT75C1* from *Arabidopsis* (Tohge et al. 2005), *AcF3GT1* from kiwifruit (Montefiori et al. 2011), and *PGT8* from *Petunia hybrida* (Yamazaki et al. 2002). Phylogenetic analysis revealed that *PpUGT73C3* groups with its allele *Ppugt73c3* and is homologous to *AtUGT75C1* from *Arabidopsis* (Fig. 5a). Notably, both proteins contain the conserved plant secondary product glycosyltransferase (PSPG) motif at the C-terminus, and the 44th amino acid of the PSPG box is a glutamine (Q) residue—a feature often associated with preference for UDP-glucose as the glycosyl donor (Fig. 5b). To assess the glycosylation catalysis activity of PpUGT73C3 and Ppugt73c3 towards cyanidin, we expressed and purified both recombinant proteins for in vitro enzyme assays. SDS-PAGE confirmed that each protein had a molecular weight of approximately 58 kDa (Fig. 5c). Functional analysis revealed that both enzymes could catalyze the formation of cyanidin-3-O-glucoside from UDP-glucose and cyanidin. Interestingly, Ppugt73c3 exhibited significantly higher enzymatic activity than PpUGT73C3 (Fig. 5d–h; Table S8).

### *PpUGT73C3* and *Ppugt73c3* positively regulate AN and PA biosynthesis in peach fruit

**Fig. 6:**
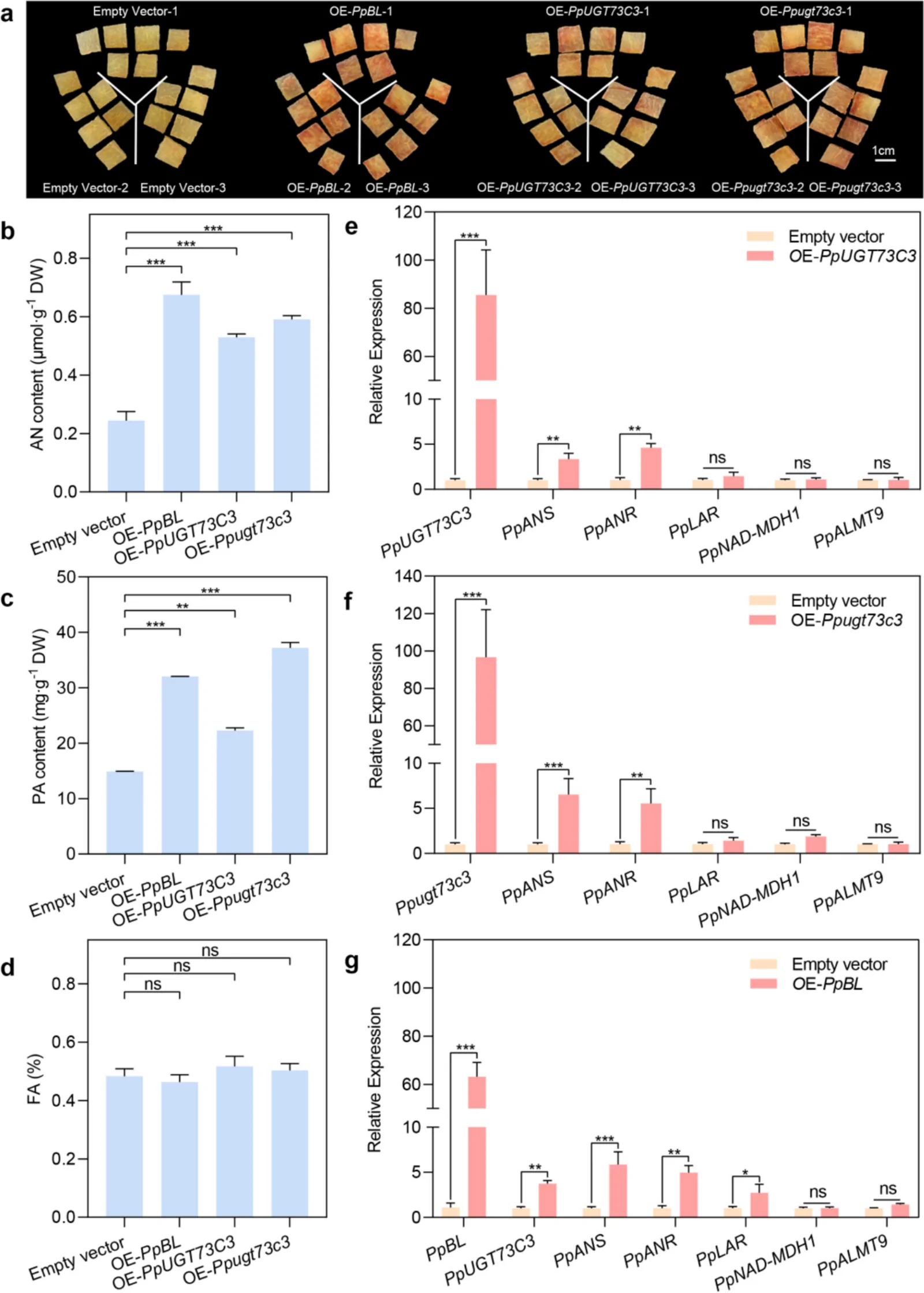
Transient overexpression of candidate genes. **a** Transient overexpression of *PpUGT73C3*, *Ppugt73c3*, and *PpBL* in white-fleshed peach. Scale bar: 1 cm. **b**–**d** AN, PA, and FA content in peach flesh infiltrated with empty vector (EV), OE-*PpBL*, OE-*PpUGT73C3*, and OE-*Ppugt73c3*. AN stands for anthocyanin, PA for proanthocyanidins, and FA for fruit acidity. Values are means ± SD for three replicates. Statistical significance: ***P < 0.001; **P < 0.01; ns, not significant. **e**–**g** Relative expression levels in peach flesh infiltrated with empty vector (EV), OE-*PpUGT73C3*, OE-*Ppugt73c3*, and OE-*PpBL*. Values are means ± SD for three replicates. Statistical significance: ***P < 0.001; **P < 0.01; ns, not significant

**Fig. 7:**
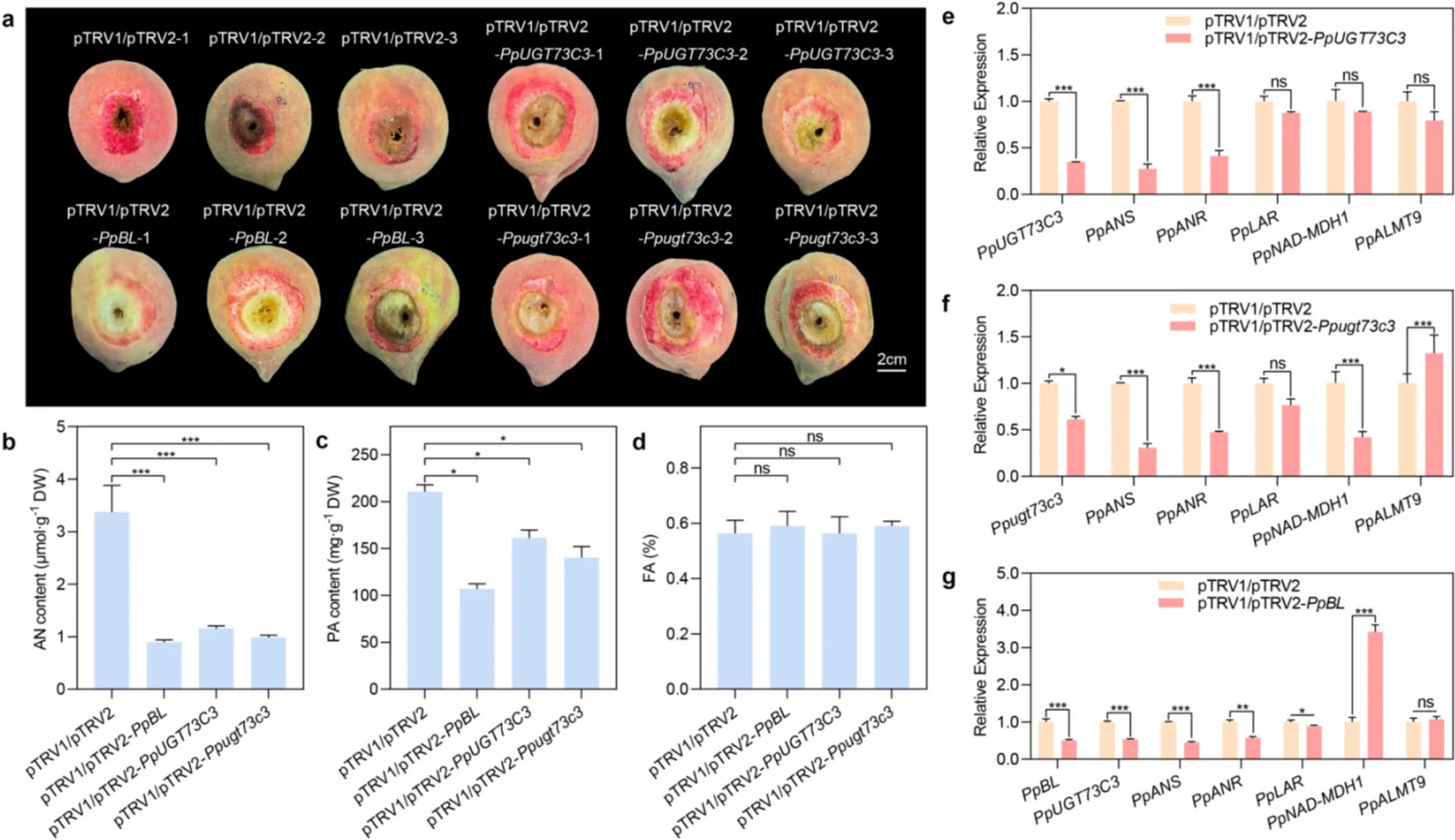
Transient silencing of candidate genes. **a** Transient silencing of *PpUGT73C3*, *Ppugt73c3*, and *PpBL* in ‘wen30’. Scale bar: 2 cm. **b**–**d** AN, PA, and FA content in peach flesh infiltrated with pTRV1/pTRV2 (empty vector), pTRV1/pTRV2-*PpBL*, pTRV1/pTRV2-*PpUGT73C3*, and pTRV1/pTRV2-*Ppugt73c3*, where AN stands for anthocyanin, PA for proanthocyanidins, and FA for fruit acidity. Values are means ± SD for three replicates. Statistical significance: ***P < 0.001; **P < 0.01; *P < 0.05; ns, not significant. **e**–**g** Relative expression levels in peach flesh infiltrated with pTRV1/pTRV2-*PpUGT73C3*, pTRV1/pTRV2-*Ppugt73c3*, and pTRV1/pTRV2-*PpBL*. Values are means ± SD for three replicates. Statistical significance: ***P < 0.001; **P < 0.01; *P < 0.05; ns, not significant

To functionally characterize *PpUGT73C3* and its allele *Ppugt73c3*, we performed overexpression and silencing assays in the white-fleshed peach ‘Datuanmilu’ and the red-fleshed peach ‘wen30’. As expected, both *PpUGT73C3* and *Ppugt73c3* induced red pigment in the pulp compared to fruits injected with empty vector (EV) as a control (Fig. 6a). Transient overexpression of *PpUGT73C3* and *Ppugt73c3* increased AN content by 2.16**-** and 2.42**-**fold, respectively, and up-regulated the expression of anthocyanin synthase (*PpANS*) (Fig. 6b, e–f). In contrast, red pigment accumulation was inhibited in the fruits injected with pTRV1/pTRV2-*PpUGT73C3* or pTRV1/pTRV2*-Ppugt73c3*, whereas it occurred normally in fruits injected with pTRV1/pTRV2 as a control (Fig. 7a). Silencing of *PpUGT73C3* and *Ppugt73c3* reduced AN content by 32% and 29%, respectively, and down-regulated the expression of *PpANS* (Fig. 7b, e–f). PA content was elevated by 1.50**-** and 2.50**-**fold in the respective overexpression lines (Fig. 6c), accompanied by enhanced expression of the key PA biosynthesis genes *PpANR* and *PpLAR* (Fig. 6e–f). Additionally, PA content was reduced by 77% and 67% in the respective silencing lines (Fig. 7c), accompanied by decreased expression of *PpANR* and *PpLAR* (Fig. 7e–f). It is worth noting that overexpression of neither *PpUGT73C3* nor *Ppugt73c3* led to significant changes in FA content, nor did it affect the expression of FA-related structural genes *PpNAD-MDH1* or *PpALMT-9* (Fig. 6d–f). Similarly, silencing of *PpUGT73C3* or *Ppugt73c3* did not cause significant changes in FA content, although their effects on the expression levels of *PpNAD-MDH1* and *PpALMT-9* were inconsistent (Fig. 7d–f).

### *PpBL* positively regulates AN and PA biosynthesis in peach fruit

Overexpression and virus-induced gene silencing (VIGS) assays in the white-fleshed peach ‘Datuanmilu’ and the red-fleshed peach ‘wen30’ were performed to verify the function of *PpBL*. Overexpressing *PpBL* enhanced red pigment accumulation and increased AN content by 2.76**-**fold compared to the control (Fig. 6a–b). qRT-PCR analysis revealed that transient overexpression of *PpBL* also up-regulated the transcript level of *PpANS* (Fig. 6g). In contrast, red pigment accumulation was inhibited in fruits injected with pTRV1/pTRV2-*PpBL*, whereas it occurred normally in fruits injected with pTRV1/pTRV2 as a control (Fig. 7a). Silencing of *PpBL* reduced AN content by 26% and down-regulated the expression of *PpANS* (Fig. 7b, g). Overexpression of *PpBL* in the pulp led to a 2.15**-**fold increase in PA content compared to the control, while PA content was reduced by 51% in the respective silencing lines (Figs. 6c; 7c). Notably, *PpBL* overexpression did not alter FA content or the expression of the FA-related structural genes *PpNAD-MDH1* or *PpALMT-9* (Fig. 6d, g). Silencing of *PpBL* also did not cause significant changes in FA content, although its effects on the expression levels of *PpNAD-MDH1* and *PpALMT-9* were inconsistent (Fig. 7d, g).

### *PpBL* directly targets *PpUGT73C3*, *PpANR*, and *PpLAR*

**Fig. 8:**
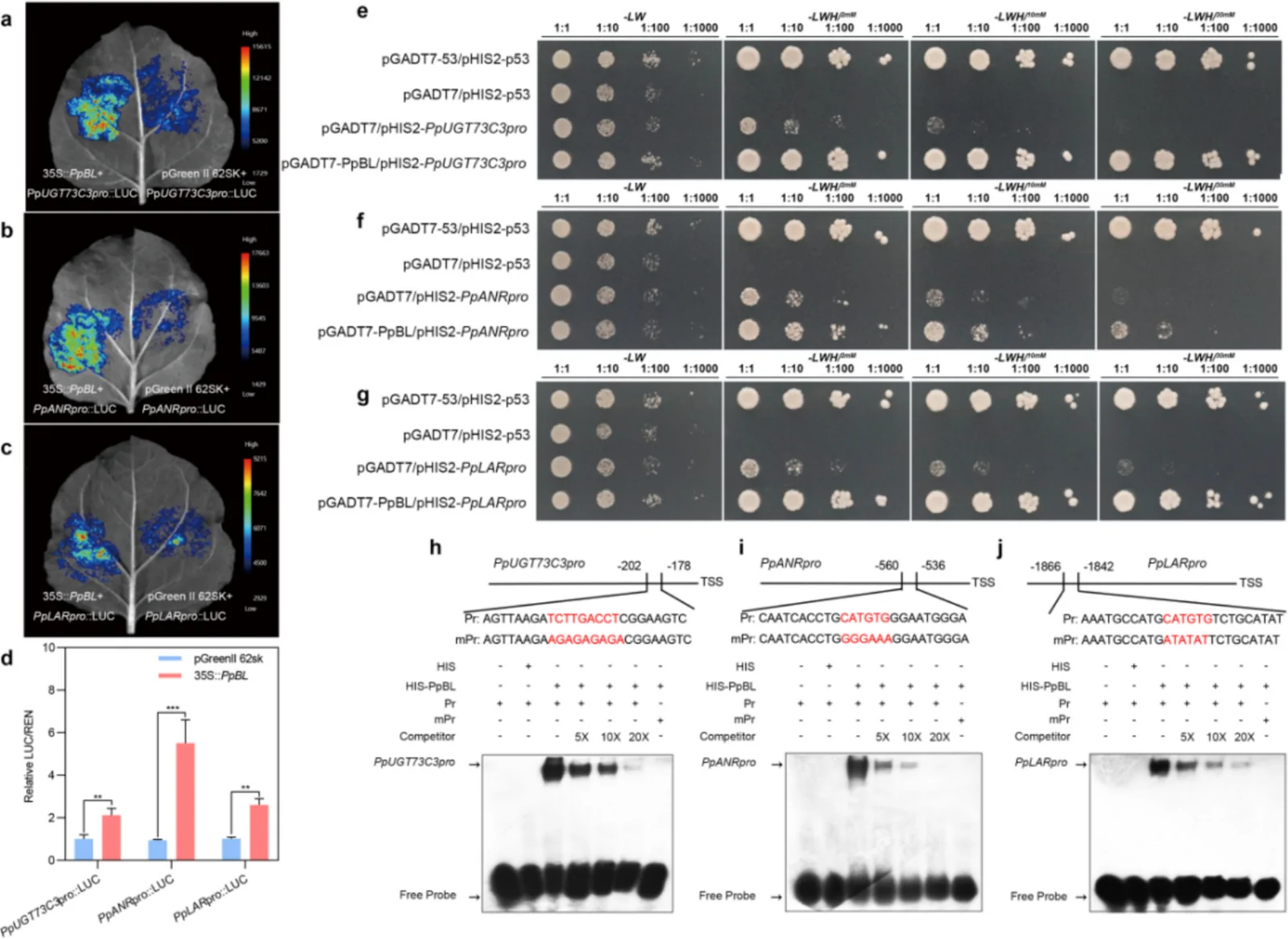
*PpBL* upregulates the expression of *PpUGT73C3*, *PpANR*, and *PpLAR* by binding to cis-acting elements in their promoters. **a**–**c** Images of *N. benthamiana* leaves were captured 3 days after infiltration. Blue and red represent low and high luminescence intensity, respectively. **d** Dual-luciferase reporter assay of PpBL on the promoter activity of *PpUGT73C3*, *PpANR*, and *PpLAR*. Values are means ± SD for three replicates. Statistical significance: ***P < 0.001; **P < 0.01. **e**–**g** Yeast one-hybrid (Y1H) assay between PpBL and the *PpUGT73C3*, *PpANR*, and *PpLAR* promoters. pGADT7-53/pHIS2-p53 and pGADT7/pHIS2-p53 were used as positive and negative controls, respectively. **h–j** Electrophoretic mobility shift assay (EMSA) showing the direct binding of His-PpBL recombinant protein to the cis-acting elements in the *PpUGT73C3*, *PpANR*, and *PpLAR* promoters. A mutated probe (mPr) was used as a control

Transient overexpression and silencing of *PpBL* not only increased and decreased AN and PA contents, respectively, but also correspondingly elevated and reduced the expression levels of *PpUGT73C3*, *PpANR*, and *PpLAR*, suggesting that *PpBL* may regulate the transcription of these genes*.* To test this hypothesis, we performed a dual-luciferase reporter assay (LUC) in *Nicotiana benthamiana* leaves. Co-injection of the effector construct *PpBL*−62SK with *PpUGT73C3pro*::LUC significantly enhanced the fluorescence signal. The LUC/REN ratio was 2.10-fold higher than that of the control (Fig. 8a, d). To identify the binding site of PpBL in the *PpUGT73C3* promoter, we analyzed its promoter sequence and identified a W-box element located 186 bp upstream of the transcription start site. The yeast one-hybrid (Y1H) assay showed that the Y187 yeast strain co-transformed with pGADT7-*PpBL* and pHIS2-*PpUGT73C3pro* grew on selective medium containing 30 mM 3-AT, whereas the control strain (pGADT7 empty + pHIS2-*PpUGT73C3pro*) did not, indicating a possible interaction between PpBL and *PpUGT73C3* promoter (Fig. 8e). Electrophoretic mobility shift assay (EMSA) demonstrated direct binding of PpBL to the W-box of *PpUGT73C3* promoter. A mutated probe, in which the W-box sequence 5'-TCTTGACCT-3' was replaced by 5'-AGAGAGAGA-3', was used as a negative control (Fig. 8h). Meanwhile, the activation of PpBL on the promoters of *PpANR* and *PpLAR* was verified by LUC, Y1H, and EMSA assays. Co-injection with *PpBL*−62SK enhanced the fluorescence intensities of *PpANRpro*::LUC and *PpLARpro*::LUC compared to the control (Fig. 8b–c). The LUC/REN ratios increased by 5.5-fold and 2.6-fold, respectively (Fig. 8d). Y1H and EMSA assays further demonstrated that PpBL binds to the E-box elements in the *PpANR* and *PpLAR* promoters (Fig. 8f–g, i–j)*.* Taken together, these results indicate that *PpBL* activates *PpUGT73C3*, *PpANR*, and *PpLAR* expression by binding to the cis-acting elements in their promoters.

## Discussion

Color is a crucial biological trait in fruit, with red-fleshed peaches commanding significant market appeal due to their vibrant pigmentation and high levels of health-promoting antioxidants, such as AN and PA (Köpsel et al. 2024; Zhao et al. 2024). Despite this appeal, red-fleshed varieties occupy only a niche segment of the peach industry, largely due to their frequently inferior flavor profiles, which include lower volatile compound content, pronounced acidity, and astringency (Yan et al. 2014; Xin et al. 2018; Chen et al. 2025). While previous studies have predominantly focused on fruit color, the flavor quality of red-fleshed peaches remains comparatively understudied (Zhao et al. 2020; Xu et al. 2024). To address this gap, we utilized an F_1_ hybrid population derived from the red-fleshed parents ‘wen30’ and ‘wen48’ to investigate the relationship between AN and key flavor compounds. By integrating QTL mapping with transcriptome data, we aimed to identify key genes coordinating color and flavor regulation in red-fleshed peaches.

Phenotypic analysis revealed significant correlations among AN, PA, and FA content, suggesting a genetic basis linking the red-flesh trait to flavor. QTL mapping identified two AN-associated loci. One locus on Chr5 was stable across two years and co-localized with a previously reported interval (Shen 2013; Cao et al. 2024) containing the NAC transcription factor *PpBL*. The other, *qAN-4* on Chr4, was detected only in 2021 and contained *Prupe.4G117900*, which encodes a glycosyltransferase homologous to *FaGT6,* a known regulator of flavonoid metabolism (Griesser et al. 2008)—making it a compelling candidate for further functional study. For PA, multiple stable QTLs were identified across several chromosomes (Ding 2017); Cao et al. 2019). Notably, the newly discovered *qPA-5* locus on Chr5 is distinct from previously reported PA QTLs, indicating a novel genetic determinant for PA content. A consistent QTL for FA was also mapped to the proximal region of Chr5, aligning with prior studies (Cao et al. 2019; Wang et al. 2022). The co-localization of AN, PA, and FA QTLs within this region reveals a specific molecular-level genetic association between color and flavor. Furthermore, the reported proximity of QTLs for volatile compounds to this region (Sánchez et al. 2014; Shi et al. 2020) suggests its potential as a key genomic hub for peach fruit quality.

Integrating QTL mapping and RNA-seq data identified two key candidate genes, *PpBL* and *PpUGT73C3*. UGT-mediated glycosylation is crucial for AN metabolism, enhancing structural diversity, stability, and solubility (Tanaka et al. 2009; Welch et al. 2008). Although many UGTs involved in AN glycosylation have been identified in plants, our enzyme assays showed that both PpUGT73C3 and its natural variant Ppugt73c3 catalyze the formation of cyanidin-3-O-glucoside from cyanidin, with Ppugt73c3 exhibiting consistently higher catalytic efficiency. Transient overexpression and VIGS assays in fruit confirmed that both versions enhance AN accumulation, with *Ppugt73c3* again showing superior efficacy. These results indicate that natural sequence variations in *PpUGT73C3*, rather than its expression level alone, influence AN content. Although *PpBL* was previously reported to activate AN synthesis via *PpMYB10.1*, our transient expression assays showed that it upregulates *PpUGT73C3* rather than Pp*MYB10.1* (Fig. S5). LUC, Y1H, and EMSA assays further demonstrated that *PpBL* directly activates *PpUGT73C3* expression. These findings point to an alternative, *PpMYB10.1*-independent pathway through which *PpBL* promotes AN accumulation.

We also found that *PpUGT73C3* promotes PA accumulation by upregulating *PpANR* expression. This is consistent with reports of UGTs enhancing PA synthesis in other species, such as *UGT72L1* from *Medicago* and a citrus UGT gene (Pang et al. 2013; Rao et al. 2019), although the precise mechanism remains unclear. Furthermore, fruit overexpression of *PpBL* increased PA content and elevated *PpANR* and *PpLAR* expression, whereas silencing of *PpBL* decreased PA content and down-regulated these genes. As demonstrated by LUC, Y1H, and EMSA assays, *PpBL* activates *PpANR* and *PpLAR* promoters, suggesting that *PpBL* contributes to PA accumulation by directly upregulating these key biosynthesis genes.

Using Kompetitive Allele Specific PCR (KASP) genotyping and resequencing data, we investigated the natural diversity of *PpBL* and *PpUGT73C3* in the F1 population. The *PpBL* promoter contains the previously reported blood-TE insertion, while the *PpUGT73C3* CDS harbors three non-synonymous SNPs. Based on these variations, the progeny were classified into four haplotypes. Hap1, which carries both the blood-TE and the non-synonymous SNPs, exhibited significantly higher AN and PA contents than haplotypes with only one or neither variation. Previous studies have shown that the blood-TE enhances *PpBL* promoter activity. To explore the dosage effect of *PpBL* on AN accumulation, we randomly selected 10 individuals without the blood-TE insertion, 10 with the heterozygous insertion, and 10 with the homozygous insertion from the F1 population, and determined relative expression level of *PpBL*. *PpBL* expression was significantly higher in individuals harboring the blood-TE than in those without the insertion. Moreover, expression levels were markedly higher in homozygous individuals than in heterozygous individuals (Fig. S6). These results demonstrate that the blood-TE acts in a dosage-dependent manner to modulate *PpBL* expression, thereby influencing downstream traits such as AN and PA content.

Notably, transient overexpression or silencing of *PpBL*, *PpUGT73C3*, or *Ppugt73c3*, significantly affected FA content, although their effects on FA-related genes *PpNAD-MDH1* and *PpALMT-9* were inconsistent, contrasting with previous reports (Chen et al. 2025). Although *PpTST1* is located within the QTL interval and is a known regulator of low fruit acidity, no expression difference was observed between red and non-red varieties. This finding is consistent with previous reports that *PpTST1* is highly expressed during late fruit development in both low-acid and high-acid varieties. A single nucleotide mutation in its coding region reduces fruit acidity, although the underlying mechanism remains unclear. Notably, *PpBL* and *PpTST1* are physically adjacent, and there is a strong genetic linkage (r = −0.84, *P* < 0.01, Table S7) between the blood-TE in *PpBL* promoter and the key SNP (G-A, Chr5: 720,760) in *PpTST1* exon, which is strongly associated with organic acid content. This tight linkage between the red-flesh color and high acidity poses a significant challenge for breeding low-acid, red-fleshed peach varieties.

## Conclusion

**Fig. 9:**
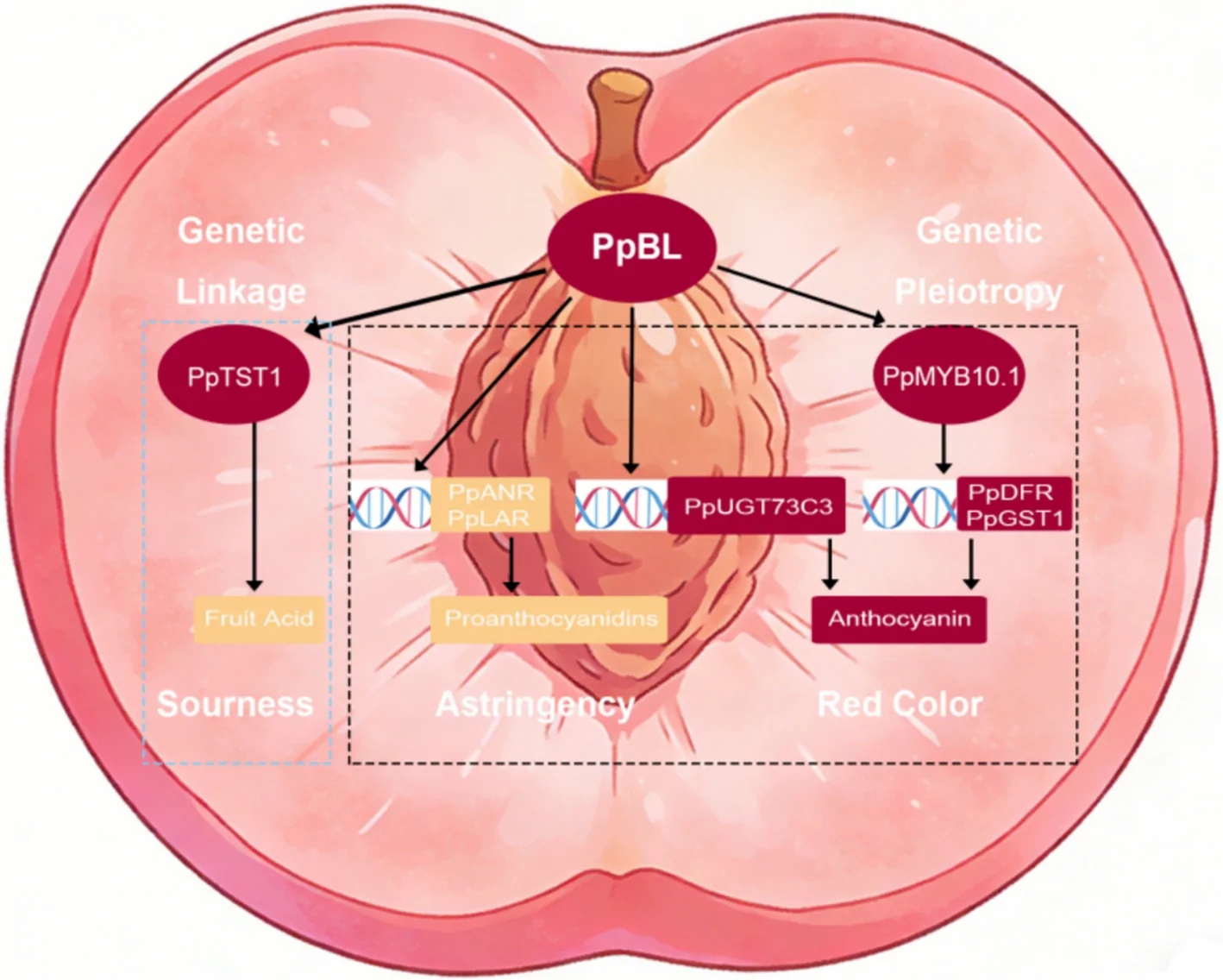
A proposed model of *PpBL* as a dual regulator of color and proanthocyanidins with tight genetic linkage to the acidity locus in red-fleshed peach. Black dashed box: pleiotropy of PpBL (color–astringency co-segregation). Blue dashed box: genetic linkage between *PpBL* and PpTST1 (color–acidity co-segregation)

This study demonstrates a significant positive correlation and genetic co-localization among AN, PA, and FA on Chr5 (3.7 cM–13.6 cM) in red-fleshed peach. We identified *PpBL* as a key regulator that enhances AN biosynthesis via *PpUGT73C3* and PA biosynthesis via *PpLAR/PpANR*. Additionally, *PpUGT73C3* also promotes PA accumulation, though the underlying mechanism remains unclear. Thus, the co-segregation of color and astringency arises from *PpBL* pleiotropy. Haplotype analysis based on a *PpBL* promoter transposon (blood-TE) and *PpUGT73C3* coding mutations reveals that hap1 (carrying both variants) confers superior AN and PA accumulation, but also higher FA due to linkage drag, underscoring its breeding value despite this trade-off. Finally, we reveal the genetic basis for the red flesh-high acidity association, demonstrating that *PpBL* does not directly regulate FA but is linked to a high-acid *PpTST1* allele via the blood-TE. Thus, the co-localization of color and acidity QTLs on Chr5 is a case of genetic linkage rather than pleiotropy. A proposed model summarizing these findings is presented in Fig. 9.

## Materials and methods

### Plant materials

A total of 152 F1 individuals derived from a cross between two red-fleshed peach cultivars, ‘wen30’ and ‘wen48’, were used for phenotypic investigation and QTL mapping. These individuals were planted in 2017, with fruit phenotypic data collected during 2021 to 2022.

Fruit development was monitored at five stages: 40, 70, 80, 90, and 100 days after blooming (s1-s5), comparing white-fleshed (WF) and red-fleshed (RF) types within the population. Transcriptome sequencing was performed on fruit samples from s3 to s5, while fruits from the white-fleshed cultivar ‘Datuanmilu’ were used for the transient transformation assay.

The ‘Datuanmilu’ materials were maintained at the National Peach Germplasm Resource Nursery of the Zhengzhou Fruit Research Institute, Chinese Academy of Agricultural Sciences (Zhengzhou, China), whereas all other experimental materials were cultivated at Xinxiang Experimental Base (Xinxiang, China).

### Phenotypic data collection

Fruit mesocarp tissues were collected, immediately frozen in liquid nitrogen, and lyophilized using a CHRIST Alpha 2–4 LDplus at −80 ℃ and 0.001 MPa until a constant weight was achieved. AN content was quantified with a Plant Anthocyanin Content Assay Kit (Boxbio, Beijing, China) following the manufacturer's protocol, and calculated according to the formula provided by the kit. Results are expressed in μmol·g^−1^ DW.

PA contents were determined using the Oligomeric Proanthocyanidins (OPC) Content Assay Kit (Boxbio, Beijing, China) according to the manufacturer's protocol. PA concentration was calculated based on the standard curve equation Y = 0.087X + 0.0196 (R^2^ = 0.997) and expressed in mg·g^−1^ DW.

FA content (%) was measured using an ATAGO digital sugar-acid refractometer PAL-B|ACID 11 (Plum). The obtained acidity value was converted to malic acid equivalents, as malic acid is the predominant organic acid in peach.

### Resequencing

Genomic DNA was extracted from fresh leaves using the CTAB method (Murray and Thompson 1980). Sequencing libraries were prepared with the GenoBaits® DNA Library Prep Kit for ILM and sequenced in PE150 mode on the MGI-2000/MGI-T7 platform (Molbreeding, Shijiazhuang, China).

Raw reads were subjected to quality control to obtain clean reads, which were then aligned to the peach reference genome v2.0 (Verde et al. 2017) using BWA (version 0.7.12). The resulting alignment files were converted to BAM format using SAMtools v1.3.1. SNP calling was performed using the Genome Analysis Toolkit v3.4 (GATK). Subsequently, population SNPs were filtered using the following criteria: minor allele frequency (MAF) < 0.05, minimum quality score (Min Q) < 30, maximum missing rate > 25%, and removal of non-dimorphic SNP loci, to obtain a set of high-quality population SNP markers.

### Construction of genetic map and QTL analysis

A high-density genetic map was constructed using Lep-MAP3 (Rastas 2017). First, the ParentCall2 function was used to obtain marker information for mapping. Second, the Separate Chromosomes2 function was used to group markers into linkage groups. With an LOD threshold of 15, the SNP markers were assigned to 8 linkage groups, corresponding exactly to the 8 chromosomes of the peach genome. Third, the JoinSingles2All function was applied to reassign ungrouped single markers into the established linkage groups. Finally, the OrderMarkers2 function was used to calculate the genetic distances within each linkage group, yielding a high-density genetic map of the population.

Since both parental lines originated from the same clonal population, GACD v1.2 software was used for QTL mapping (Zhang et al. 2015). QTL analysis was performed using the ICIM-ADD mapping method with default parameters (PIN = 0.001, walking step = 1.0 cM). Significance thresholds were determined by 1,000 permutation tests (type I error = 0.05). The QTL confidence interval was the mapping interval corresponding to the decrease in LOD on either side of the peak. QTLs were named starting with ‘q’, followed by the trait abbreviation and the chromosome number. QTL mapping results were visualized using MapChart (Voorrips 2002).

### Transcriptome analysis

Total RNA was extracted using TRIzol reagent (Invitrogen, Carlsbad, CA, USA). After quality control, libraries were constructed and subjected to paired-end sequencing on an Illumina platform. Clean reads obtained from raw data were aligned to the peach reference genome v2.0 (Verde et al. 2017) using HISAT2. Read Counts and FPKM values for each sample were calculated using featureCounts (v2.0.3). Differentially expressed genes (DEGs) were identified using the R package DESeq2, with screening thresholds set at |log_2_(Fold Change)|≥ 1 and adjusted *P*-value (padj) < 0.05.

### Gene expression analysis

Quantitative real-time PCR (qRT-PCR) was performed to analyze gene expression levels using LightCycler 480 System (Roche Diagnostics) according to the manufacturer's protocol. Relative expression levels were calculated via the 2^−△△Ct^ method. The primers were designed with Primer-BLAST software and are listed in Supplementary Table S1.

### In vitroassay of recombinant* PpUGT73C3 *enzyme

The CDS of *PpUGT73C3* and *Ppugt73c3* were separately cloned into the pET-28a expression vector. The recombinant proteins with an N-terminal 6 × His tag were purified via Ni^2+^ affinity chromatography (GE Healthcare, Chicago, IL, USA). Enzyme activity was determined following a previously described method with slight modifications (Yan et al. 2025). The reaction mixture consisted of 300 μL of UDP-glucose (1 mg/mL), 100 μL of cyanidin (0.5 mg/mL), and 20 μg of recombinant protein, with the final volume adjusted to 600 μL using 0.1 mol/L Tris–HCl buffer (pH 7.8). After incubation for 5 min, the reaction was terminated by adding 100 μL of HCl solution. The mixture was centrifuged at 12,000 × g for 5 min, and the resulting supernatant was collected for product detection using an Agilent 1290–6460 LC–MS/MS system (Agilent Technologies, Santa Clara, CA, USA).

### Transient overexpression assay in peach fruit

The CDS of *PpBL*, *PpUGT73C3*, and *Ppugt73c3* were cloned into the pBI121 vector downstream of the CaMV 35S promoter to generate overexpression constructs. The empty pBI121 vector and the constructs were then introduced into *Agrobacterium tumefaciens* strain GV3101. White-fleshed ‘Datuanmilu’ fruits were selected for the transient transformation assay. Following agroinfiltration, the fruits were cultured on MS medium supplemented with 100 mg/L kanamycin and 300 mg/L timentin, and kept in darkness for 24 h followed by 48 h under light conditions, according to the method described by Liu et al. (2017).

### Virus-induced gene silencing (VIGS) in peach fruit

VIGS experiments targeting *PpBL*, *PpUGT73C3*, and *Ppugt73c3* in peach fruit were conducted according to a previously described method (Peng et al. 2020). ‘wen30’ fruits were selected for the VIGS assay, and fruits injected with the empty vector (pTRV1/pTRV2) were used as the negative control.

### Dual-luciferase (LUC) assay in tobacco leaves

The promoters of *PpUGT73C3*, *PpANR*, and *PpLAR* (2 kb upstream of the transcription start site) were amplified and inserted into the pGreenII 0800-LUC vector upstream of the *LUC* reporter gene (Hellens et al. 2005). The CDS of *PpBL* was cloned into the pGreenII 62-SK vector downstream of the CaMV 35S promoter. For agroinfiltration, the infiltration solution containing the pGreenII 0800-LUC recombinant vector was mixed with that containing the pGreenII 62-SK construct at a 1:5 volume ratio and kept in the dark for 2 h. The mixed suspension was then infiltrated into *Nicotiana benthamiana* leaves*.* After infiltration, the leaves were incubated in darkness for 16**–**24 h, followed by 24 h under light. Leaf discs were soaked in Dual Luciferase Sodium Salt Buffer (Solarbio, Beijing, China) for 10 min before imaging to quantify LUC intensity. The LUC/REN ratio was measured using the Dual Luciferase Reporter Gene Assay Kit (YEASEN, Shanghai, China). At least 3 biological replicates were performed for each combination.

### Yeast one-hybrid (Y1H) assay

The CDS of *PpBL* was cloned into the pGADT7 vector to generate pGADT7-*PpBL*. The promoters of *PpUGT73C3*, *PpANR*, and *PpLAR* were cloned into the pHIS2 vector to generate pHIS2-*PpUGT73C3pro*, pHIS2-*PpANRpro*, and pHIS2-*PpLARpro*. The plasmids pGADT7-*PpBL*, pHIS2-*PpUGT73C3pro*, pHIS2-*PpANRpro*, and pHIS2-*PpLARpro* were co-transformed into the yeast strain Y187. pGADT7-53/pHIS2-p53 and pGADT7/pHIS2-p53 were used as positive and negative controls, respectively. In addition, pGADT7 together with pHIS2-*PpUGT73C3pro*, pHIS2-*PpANRpro*, or pHIS2-*PpLARpro* was used as self-activation controls. The primers used for Y1H are listed in Table S1.

### Electrophoretic mobility shift assay (EMSA)

For EMSA, the recombinant PpBL protein was expressed in prokaryotic cells. The fusion protein was purified using STarm Streptactin Beads 4FF (Smart-Lifescience, Changzhou, China), and the quality of the recombinant protein was assessed by SDS-PAGE. Biotin-labeled oligonucleotide probes for the promoters of *PpUGT73C3*, *PpANR*, and *PpLAR* were synthesized by PCR. EMSA was performed using the LightShift® Chemiluminescent EMSA Kit (Thermo Fisher Scientific, Waltham, MA, USA), and the probes used in this study are listed in Table S1.

### Identification of blood-TE

The Kompetitive Allele Specific PCR (KASP) assay was performed according to the previous description (Meng et al. 2021). The instrument used was the LightCycler 480 (LC480); the KASP enzyme was FLU-ARMS for KASP 2 × PCR Mix (Good Biotech, Guangzhou, China). The reaction system contained: 5 μL of Mix, 0.1 μL each of FAM and HEX primers, 0.3 μL of common primer, 3.5 μL of ddH_2_O and 1 μL of template. Corresponding primer sequences were designed using Primer-BLAST and are provided in Supplementary Table S1.

### Data analysis

Data analysis was performed using Microsoft Excel 2021. Correlations among fruit agronomic traits were examined using the *corrplot* package in R. Additionally, the broad-sense heritability (H^2^) was computed using the analysis of variance (ANOVA) function of QTL IciMapping.

## Supplementary Information


Additional file 1: Fig. S1. The high-density genetic map of ‘wen30’ and ‘wen48’ F_1_ population. Fig. S2. Summary of QTLs identified in the study. a QTLs for anthocyanin (AN). b QTLs for proanthocyanin (PA). c QTLs for fruit acidity (FA). Fig. S3 The differentially expressed genes (DEGs) between white-fleshed (WF) and red-fleshed (RF) in s3-s5. a Color changes of WF and RF during stages s1 to s5. Scale bar: 2cm. b The upregulated DEGs between WF and RF in s3-s5. c The downregulated DEGs between WF and RF in s3-s5. Fig. S4 The identification of blood-TE in *PpBL* promoter. Fig. S5 Relative expression level of *PpMYB10.1* in the pulp of transient *PpBL*. a. Relative expression level of *PpMYB10.1* in the pulp of transient overexpression *PpBL*. Values are means ± SD for three replicates. Statistical significance: ns, no significance. b. Relative expression level of *PpMYB10.1* in the pulp of transient silence *PpBL*. Values are means ± SD for three replicates. Statistical significance: ns, no significance. Fig. S6 The relative expression of *PpBL *in different samples. The blood‑TE −/− indicates the absence of blood‑TE insertion, blood‑TE −/+ indicates heterozygous insertion, blood‑TE +/+ indicates homozygous insertion.Additional file 2: Table S1. Primers in this study. Table S2. Sequencing data statistics for all the samples. Table S3 QTLs for AN, PA and FA in the ‘wen30’ and ‘wen48’ F1 population. Table S4. The AN, PA content and FA of WF and RF peaches in s1-s5. Table S5. RNA-seq data statistics. Table S6. Candidate genes identified by both QTL and RNA-seq. Table S7. Genotype diversity of the two parents and F1 population. Table S8. *In vitro* assay of the recombinant PpUFT73C3 activities.

## Data Availability

The authors confirm that all the experimental data is available and accessible via the main text and/or the supplemental data.

## Abbreviations

AN: Anthocyanin
CDS: Coding sequence
DEGs: Differentially expressed genes
EMSA: Electrophoretic mobility shift assay
FA: Fruit acid
GWAS: Genome-wide association studies
H^2^: Broad-sense heritability
KASP: Kompetitive Allele Specific PCR
LUC: Dual-luciferase reporter assay
PA: Proanthocyanidins
PSPG: Plant secondary product glycosyltransferase (conserved motif of UGTs)
PVE: Phenotypic variance explained
QTL: Quantitative trait locus
RT-qPCR: Quantitative real-time PCR
VIGS: Virus-induced gene silencing
Y1H: Yeast one-hybrid
