# Supplementary material for: Dual regulation of anthocyanins and proanthocyanidins by PpBL and its linkage to acidity in peach
Source: Mol Hortic. 2026 Sep 1;6:67. doi: 10.1186/s43897-026-00255-6 (PMC13531910; doi:10.1186/s43897-026-00255-6)
Supplement: Supplementary file 1 [file 43897_2026_255_MOESM1_ESM.docx]

Fig. S1 High-density genetic map of the 'wen30' × 'wen48' F1 population.

Fig. S2 Summary of QTLs identified in this study. a QTLs for anthocyanin (AN). b QTLs for proanthocyanidins (PA). c QTLs for fruit acidity (FA)..

Fig. S3 Differentially expressed genes (DEGs) between white-fleshed (WF) and red-fleshed (RF) peaches at stages s3 to s5. a Color changes of WF and RF during stages s1 to s5. Scale bar: 2 cm. b The upregulated DEGs between WF and RF in s3-s5.c The downregulated DEGs between WF and RF at stages s3 to s5.

Fig. S4 Identification of blood-TE in the *PpBL* promoter..

Fig. S5 Relative expression levels of *PpMYB10.1* in peach pulp following transient *PpBL* modulation. a Relative expression level of *PpMYB10.1* in the pulp after transient overexpression of *PpBL*. Values are means ± SD for three replicates. Statistical significance: ns, not significant. b Relative expression level of *PpMYB10.1* in the pulp after transient silencing *PpBL*. Values are means ± SD for three replicates. Statistical significance: not significant.

Fig. S6 Relative expression of *PpBL* in different samples. The blood‑TE −/− indicates the absence of blood‑TE insertion, blood‑TE −/+ indicates heterozygous insertion, blood‑TE +/+ indicates homozygous insertion.
